# Epigenetic Changes Induced by High Glucose in Human Pancreatic Beta Cells

**DOI:** 10.1155/2023/9947294

**Published:** 2023-02-13

**Authors:** Rasha A. Alhazzaa, Raechel E. McKinley, Bruk Getachew, Yousef Tizabi, Thomas Heinbockel, Antonei B. Csoka

**Affiliations:** ^1^Department of Anatomy, Howard University, 520 W St. NW, Washington DC 20059, USA; ^2^King Saud bin Abdulaziz University for Health Sciences, Riyadh 14611, Saudi Arabia; ^3^Department of Pharmacology, Howard University, 520 W St. NW, Washington DC 20059, USA

## Abstract

Epigenetic changes in pancreatic beta cells caused by sustained high blood glucose levels, as seen in prediabetic conditions, may contribute to the etiology of diabetes. To delineate a direct cause and effect relationship between high glucose and epigenetic changes, we cultured human pancreatic beta cells derived from induced pluripotent stem cells and treated them with either high or low glucose, for 14 days. We then used the Arraystar 4x180K HG19 RefSeq Promoter Array to perform whole-genome DNA methylation analysis. A total of 478 gene promoters, out of a total of 23,148 present on the array (2.06%), showed substantial differences in methylation (*p* < 0.01). Out of these, 285 were hypomethylated, and 193 were hypermethylated in experimental vs. control. Ingenuity Pathway Analysis revealed that the main pathways and networks that were differentially methylated include those involved in many systems, including those related to development, cellular growth, and proliferation. Genes implicated in the etiology of diabetes, including networks involving glucose metabolism, insulin secretion and regulation, and cell cycle regulation, were notably altered. Influence of upstream regulators such as MRTFA, AREG, and NOTCH3 was predicted based on the altered methylation of their downstream targets. The study validated that high glucose levels can directly cause many epigenetic changes in pancreatic beta cells, suggesting that this indeed may be a mechanism involved in the etiology of diabetes.

## 1. Introduction

Diabetes mellitus (DM) is a chronic metabolic disease in which either the pancreas produces very little or no insulin, or the target cells of the body do not respond correctly to the insulin produced, resulting in high levels of glucose in the blood and urine. DM is associated with both genetic and environmental factors. The cost of treating it and its complications has considerably increased because of an epidemic of DM worldwide [1].

Epigenetics is a term used to describe heritable (somatic or meiotic) changes in gene expression not caused by changes in the DNA sequence, resulting in alterations in the way cells “read” or express genes. Additionally, epigenetics relates to a causal chain linking genetics, environmental exposure, and disease development. Epigenetic changes occur often during an organism's lifetime and may be transmitted to the next generation [2]. Also, numerous factors may cause epigenetic changes, such as breastfeeding and maternal care, physical activity or inactivity, hyperglycemia, mitochondrial dysfunction, aging, and menopause [2]. Epigenetic state may even be influenced by pharmaceutical drugs [3] and integrative medicine [4].

Three major epigenetic systems are the focus of current research: DNA methylation, covalent posttranslational modification to histones (i.e., methylation, phosphorylation, acetylation, and ubiquitylation), and noncoding RNAs (ncRNAs) that modify gene expression at the transcriptional and posttranscriptional stages.

Several studies have already suggested that epigenetics plays a significant role in the etiology of DM, in particular T2D [5–8]. Also, the changes in T2D, such as chronic hyperglycemia, hypoxia, increased oxidative stress, and dyslipidemia, could both result from, and cause, epigenetic alterations associated with the disease [8–10]. Furthermore, it is known that hypoxia influences several precursor cell types' stemness and lineage commitment during development, including pancreatic beta cells [11]. *In vivo*, beta cell neogenesis is negatively regulated by hypoxia-inducible factor 1 alpha (HIF-1*α*) caused by low oxygen tension [12]. It has also been found that repression of HIF-1*α* causes increased development of Ngn3-positive endocrine progenitors, resulting in higher levels of beta cell neogenesis and growth [12]. Furthermore, the deletion of the von Hippel-Lindau (VHL) gene, which encodes a protein involved in the degradation of HIF-1*α*, leads to compromised beta cell development. This will have a further negative impact on beta cell neogenesis [13]. Furthermore, Sun et al. found that hypoxia (2% pO_2_) directly affects the differentiation of mesenchymal stem cells into early beta cell progenitors [14].

The principal insulin-producing cells in the pancreas are the beta cells, and epigenetic tags play a crucial role in establishing and sustaining their functional identity [9]. Stable beta cell function is critical for the regulation of glucose levels in the blood. In contrast, epigenetic dysregulation results in decreased expression of genes essential for beta cell function, the inappropriate activation and expression of genes that should normally remain repressed, and compromised genetic imprinting, resulting in loss of beta cell identity [9] and reduced insulin secretion [6].

The origin of an altered epigenetic state in beta cells in DM has not been fully elucidated. Beta cells could be an especially sensitive target in prediabetes and related conditions, because not only do they produce insulin, but also respond to insulin and glucose levels themselves. To directly test the hypothesis that a high glucose environment, characteristic of prediabetes, can cause epigenetic changes, we wanted to establish a direct cause and effect link between high glucose levels and an altered epigenetic state in the cells. Therefore, we treated beta cells differentiated from induced pluripotent stem cells (iPSCs) with high (15 mM) vs. low (2 mM) glucose and looked for epigenetic changes by performing genome-wide DNA methylation analysis. We also investigated affected signaling pathways and gene networks, including those thought to be involved in the etiology of DM. Our hypothesis was that a high glucose concentration would cause significant genome-wide epigenetic changes in pancreatic beta cells and that this dynamic could promote the development of DM.

## 2. Materials and Methods

### 2.1. Cell Culture

For this study, Cellartis hiPS Beta Cells (Takara Bio, CA, USA) were used to investigate beta cell activity. The benefits of using these cells are that they can be conveniently used instead of pancreatic islets. In some ways, they are superior to islets because they are pure beta cells and can be used as a physiologically important model for insulin development and release. These cells secrete high amounts of insulin and C-peptide and exhibit significant protein expression of MAFA, NKX6-1, PDX1, and UCN3. Also, they are differentiated from an iPSC cell line, ChiPSC22 (Takara Bio, CA, USA), derived from a single donor.

To grow the cells, 6-well plates (Corning, USA) were coated with Cellartis Beta Cell Coating and cultured in growth medium containing Cellartis Beta Cell Basal Medium and supplemented with Cellartis Beta Cell Supplement (Takara Bio, CA, USA). Cells were incubated and maintained in 5% CO_2_ at 37°C.

### 2.2. MTT Assay

**Figure 1:**
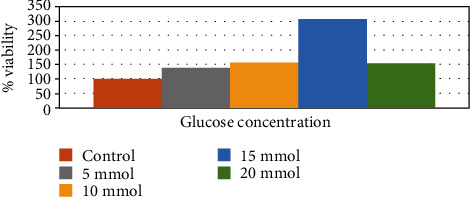
% Cell viability using MTT assay. No negative effect was observed on cell morphology or growth kinetics at or below 15 mM, but at a concentration of 20 mM, a cytotoxic effect was noted. A 15 mM solution of glucose was determined to be the maximum concentration that could be used without inducing cell death.

**Figure 2:**
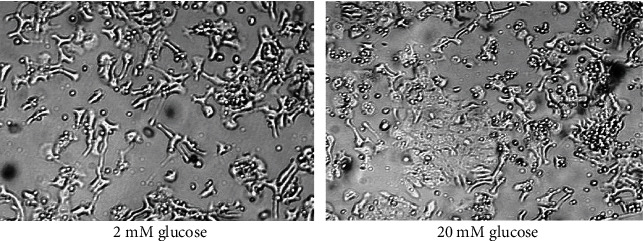
Cell morphology and cell death. Images of hiPS beta cells in concentrations of glucose of 2 mM and 20 mM. It was noticed that there was a difference in cellular morphology between the high- and low-glucose groups, indicative of increased cell death at the higher concentration.

A cell viability and toxicity curve was initially performed on the cells using an MTT assay to determine the optimum glucose concentration that can be tolerated without causing cell death. Cells were grown in growth media containing various amounts of glucose (5 mM, 10 mM, 15 mM, and 20 mM) for 48 hours. From 5 mM to 15 mM, cell proliferation increased, but at 20 mM, it decreased (Figure 1). This decrease corresponded to a visible increase in cell death (Figure 2). We observed a difference in cellular morphology between high and low glucose concentrations during this time, e.g., cell differentiation, death, and detachment. These changes are recapitulated *in vivo*; for example, after being treated with high glucose, beta cells go into neogenesis rather than proliferation. The underlying mechanism for this is an increase in insulin resistance in the beta cell mass and impaired glucose tolerance. In addition, this beta cell functional plasticity contributes to the compensation for an increased insulin demand caused by insulin resistance. The mechanism for reduced beta cell mass in T2D is likely a dramatically increased rate of apoptosis [15]. Also, short-term exposure of beta cells to increasing glucose instigates proliferation in a concentration-dependent manner. Hyperglycemia also inhibits beta cell secretory function which is evident before the apoptosis leads to the decrease in cell mass [16]. We saw all these changes occurring in parallel *in vitro*, and therefore, a 15 mM solution of glucose was the maximum concentration that could be used without inducing cell death. Notably, within the literature, insulin secretion increased significantly at 16.7 mM glucose [17].

### 2.3. Cell Treatment

The hiPS Beta Cells were thawed, resuspended in Beta Cell Maintenance medium, and plated in culture vessels coated with Beta Cell Coating. We seeded the cells into six-well plates in two groups of three: low and high glucose. Three wells served as low-glucose controls, and three (experimental) wells received high glucose. The control group was maintained throughout in the minimum glucose concentration required for cellular viability and maintenance of enrichment of beta cells (2 mM at the recommendation of Takara Bio). For the first four days, media was changed every 24 hours and all six wells contained 2 mM glucose. On days five to eight, glucose was increased to 10 mM in the three experimental wells. Then, on days nine to fourteen, we gradually increased the glucose concentration to reach 15 mM in the three experimental wells, to ensure that the cells did not go into shock or glucose toxicity (defined as a temporary physiological condition triggered by frequent or extended exposure to high glucose concentrations) [18].

### 2.4. DNA Extraction and MeDIP-Chip Analysis

After 14 days, the 2 mM or 15 mM glucose-treated cells were lysed, and genomic DNA (gDNA) was homogenized using Qiashredder (Qiagen, Fremont, CA) and extracted using the DNeasy kit (Qiagen, Fremont, CA). The DNA processing protocol then closely follows our previous paper [19]. The purified gDNA was then quantified, and quality assessed by NanoDrop ND-1000. This was followed by sonication to generate fragments of about 200-1000 base pairs. Immunoprecipitation of methylated DNA was performed using Biomag™ magnetic bead-coupled mouse monoclonal antibody against 5-methylcytidine. The immunoprecipitated DNA was eluted and purified by phenol chloroform extraction and ethanol precipitation [19]. The total input and immunoprecipitated DNA were labeled with Cy3- and Cy5-labeled random 9-mers, respectively, and hybridized to the Arraystar 4x180K HG19 RefSeq Promoter Array, which is a multiplex slide with 4 identical arrays per slide, and each array contains 23,148 well-characterized RefSeq promoter regions (from about -1,300 bp to +500 bp of the Transcription Start Sites) totally covered by ~180,000 probes. Scanning was performed by using Agilent Scanner G2505C (Rockville, MD, USA) [19].

### 2.5. Data Normalization

Again, normalization followed our previous protocol [19]. Raw data was extracted as txt files by Agilent Feature Extraction software. We performed median centering, quantile normalization, and linear smoothing by Bioconductor packages (Ringo, limma, and MEDME) [20]. The enrichment peaks and differentially methylated peaks were analyzed and annotated by NimbleScan software. The user guide and result data formats can be found at http://www.nimblegen.com/downloads/support/NimbleScan_v2p6_UsersGuide.pdf. After normalization, a normalized log2-ratio data (^∗^_ratio.gff file) was created for each sample. From the normalized log2-ratio data, a sliding-window peak-finding algorithm provided by NimbleScan v2.5 (Roche-NimbleGen) was applied to find the enriched peaks with specified parameters (sliding window width: 1,500 bp; mini probes per peak: 2; *p* value minimum cutoff: 2; maximum spacing between nearby probes within peak: 500 bp). After getting the ∗_peaks.gff files, the identified peaks were mapped to genomic feature transcripts [19].

### 2.6. Bioinformatic and Pathway Analysis of MeDIP-Chip Results

As before [19], *T*-tests and/or binomial tests were used to compute *p* values for differential methylation of CpG sites followed by multiple comparison correction of *p* values and computation of false detection ratio (FDR) using the Benjamini-Hochberg method [21]. Genes that are significantly differentially methylated (*p* < 0.01) between the treated vs. control groups were identified, and functional analysis of differentially methylated genes was performed using Gene Set Enrichment Analysis (GSEA). Gene promoters showing statistically significant changes in DNA methylation patterns were subjected to Ingenuity Pathway Analysis (IPA) (Ingenuity System Inc., CA, USA) for signaling pathway and gene network analysis. The *z* scores predict activation states of transcriptional regulators and were calculated by an IPA-based algorithm [19].

(http://pages.ingenuity.com/rs/ingenuity/images/0812%20upstream_regulator_analysis_whitepaper.pdf).

## 3. Results

### 3.1. Genome-Wide Methylation Analysis and Investigation of Pathways, Functions and Networks

The analysis of genome-wide methylation revealed that high glucose caused significant differential methylation (*p* < 0.01) in 478 gene promoters (from about -1,300 bp to +500 bp of the transcription start sites) (2.06%). There were more promoters hypomethylated (285; 1.23%) than hypermethylated (193; 0.83%) (Supplement 1a, https://docs.google.com/spreadsheets/d/10npdt2_UEH9T3gX3d3qZIkm07mf5mtJf/edit#gid=1906293732).

**Figure 3:**
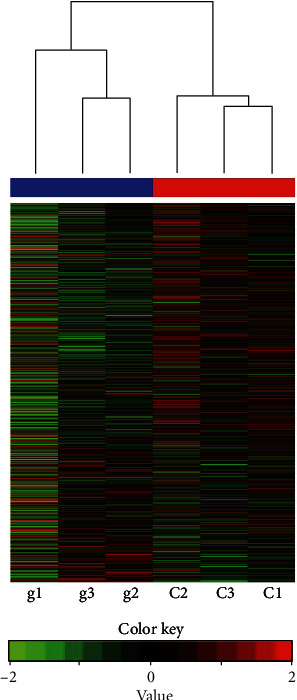
Heat map of hypermethylated and hypomethylated gene promoters. MeDIP-Chip analysis grouped into clusters shows the heat map of differentially methylated gene promoters between glucose-treated (g1, g2, and g3) and control-treated (C1, C2, and C3) samples. The scale symbolizes hypomethylated promoters (values 0 to +2) in green and hypermethylated promoters (values 0 to −2) in red. Each tiny row represents a gene on a specified sample signified by columns. There was a clear demarcation between the control samples with most of the genes that are either downregulated (hypermethylated promoters in red) or upregulated (hypomethylated promoters in green) between samples represented in the six rows. The overall methylation clustering between treated and control samples on the top (red and blue) represents quantitative methylation clustering between significant genes.

Means for all the samples can be found in Supplement 1b (https://docs.google.com/spreadsheets/d/1_SKHu_rP3wDacDzDqUUS_zN88XaEVhUx/edit#gid=1273186519). A heat map (Figure 3) represents differential DNA methylation between treated (g1, g2, and g3) and control (C1, C2, and C3), which are grouped into two clear clusters.

As before [19], since our analysis only included significant gene promoters without intragenic and intergenic regions, we could translate our methylation data into gene expression data for IPA without complication; hypermethylated promoters are representing downregulation and hypomethylated promoters are representing upregulation of gene expression, by default [19]. We assigned positive and negative values to peak differential methylation values to correlate to upregulation or downregulation of gene expression, respectively (Supplement 2, https://docs.google.com/spreadsheets/d/17tlE8xz3xbaNPrI9Iz18YZXOmjF4qlpt/edit#gid=407977637). Henceforth, we will refer to these gene promoters as genes for simplicity and refer to activation from gene induction as upregulation and inhibition from gene silencing as downregulation [19].

**Figure 4:**
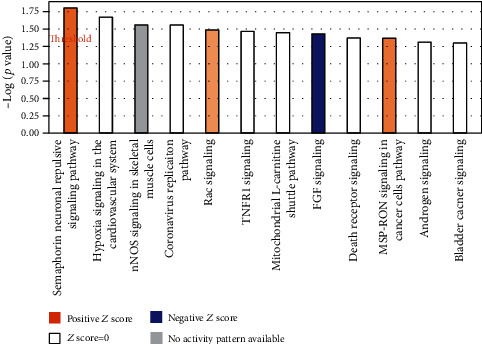
Top canonical pathways altered by glucose. The bar graph depicts the main canonical pathways predicted to be altered by high glucose treatment using IPA core analysis. High glucose causes differential methylation of significant genes from our dataset that are enriched in canonical pathways, such as semaphorin neuronal repulsive signaling pathway, hypoxia signaling in the cardiovascular system, nNOS signaling in skeletal muscle cells, coronavirus replication pathway, and Rac signaling based on their *z* score, ratio, and −log(*p* value). The threshold is set at the lowest level of confidence that is statistically acceptable (*p* ≤ 0.05).

**Figure 5:**
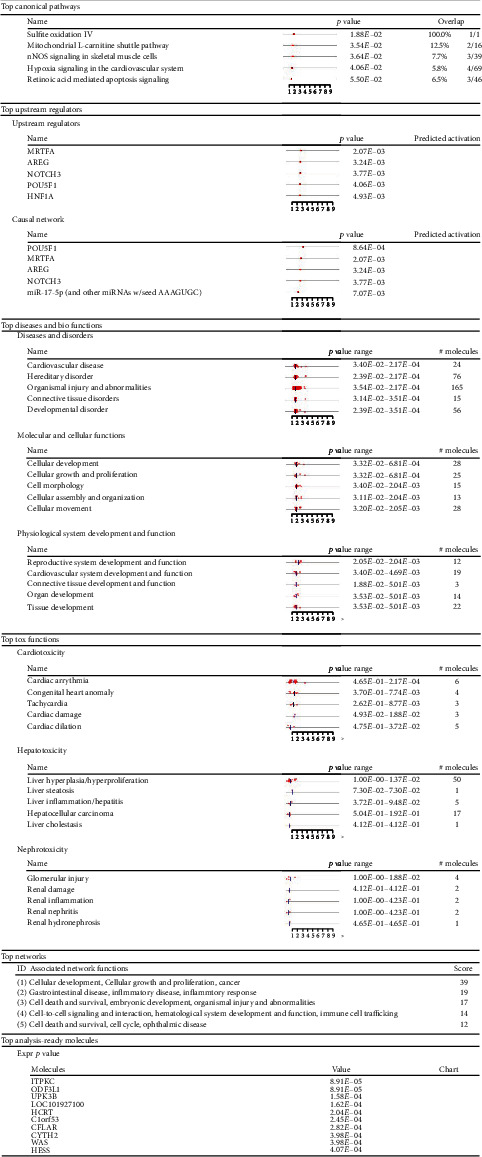
IPA analysis. An initial analysis of canonical pathways, upstream regulators, diseases and biological functions, toxicological functions, networks, and analysis-ready molecules using IPA's core analysis function.

**Figure 6:**
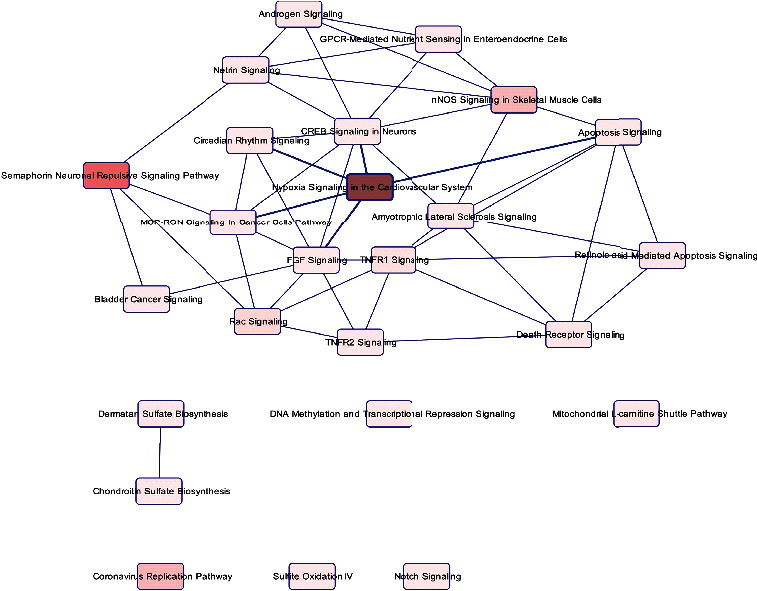
Connections between pathways. This figure shows many connected pathways, which means that most of the significantly altered genes are shared between them.

In an initial analysis of canonical signaling pathways, biological functions, and gene networks using IPA's core analysis function, we found that significant genes were enriched in diverse canonical pathways such as the semaphorin neuronal repulsive signaling pathway (*p* value = 1.57*E* − 02), hypoxia signaling in the cardiovascular system (*p* value = 2.13*E* − 02), nNOS signaling in skeletal muscle cells (*p* value = 2.76*E* − 02), the coronavirus replication pathway (*p* value = 2.76*E* − 02), and Rac signaling (*p* value = 3.24*E* − 02) (Figure 4 and Figure 5). Many of these pathways are connected (Figure 6).

**Figure 7:**
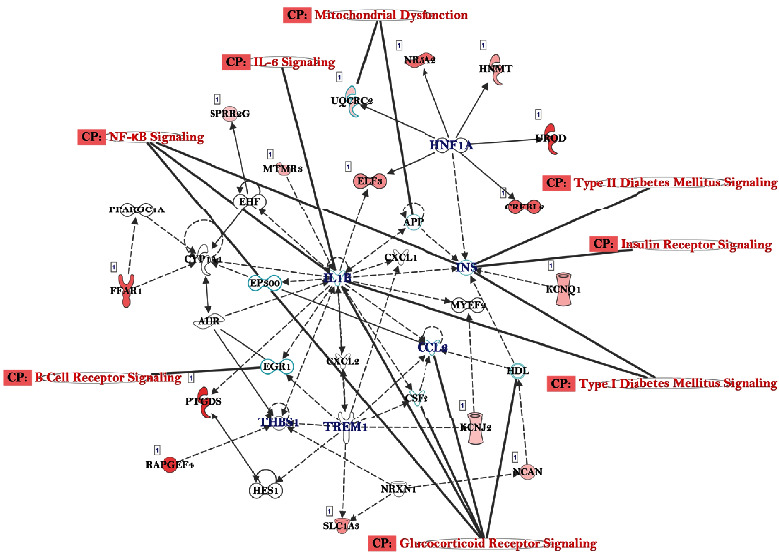
IPA analysis identifying the main networks of differentially expressed genes and canonical pathways that contribute to the etiology of DM. Genes are presented with significant effectors on expression pattern with ≥2.0 fold change and *p* value < 0.05. Dotted lines connecting the genes represent indirect connections, while the straight lines represent directly connecting genes and related pathways.

We particularly wanted to analyze any differential methylation caused by glucose in genes that are part of the glucose modifiers groups or involved in the etiology of DM, especially T2D. With further analysis using IPA, we linked to mechanistic networks and canonical pathways (Figure 7).

35 of these genes showed significant differential methylation (Supplement 3, https://docs.google.com/spreadsheets/d/1vV9kzpqkS4Zoc_4Hmi3lMrXrvZhpw19v/edit#gid=386233314), including CFLAR, WAS, TP73, and ERBB2.

**Figure 8:**
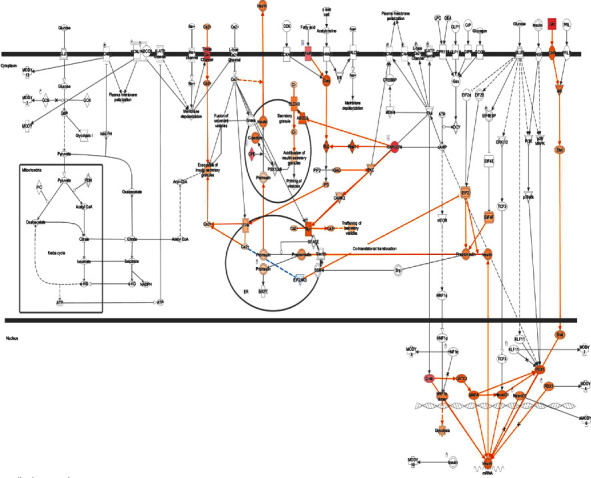
Insulin secretion signaling pathway and upregulated molecules. CPE, Creb, FFAR1, RAPGFF4, and T-Type-Ca Channel are upregulators that activate many genes, while ELF2AK3 mostly inhibits.

**Figure 9:**
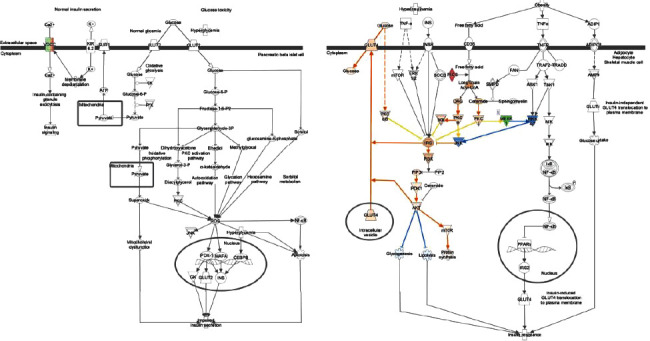
T2D signaling and upregulated molecules. GLUT4, IRS, DAG, and AKT are significant metabolic pathways that activate many genes, while MKK 4/7 and JNK play a role in inhibition.

We found many genes involved in the insulin secretion signaling pathway (Figure 8), and several genes involved in T2D signaling (Figure 9).

**Figure 10:**
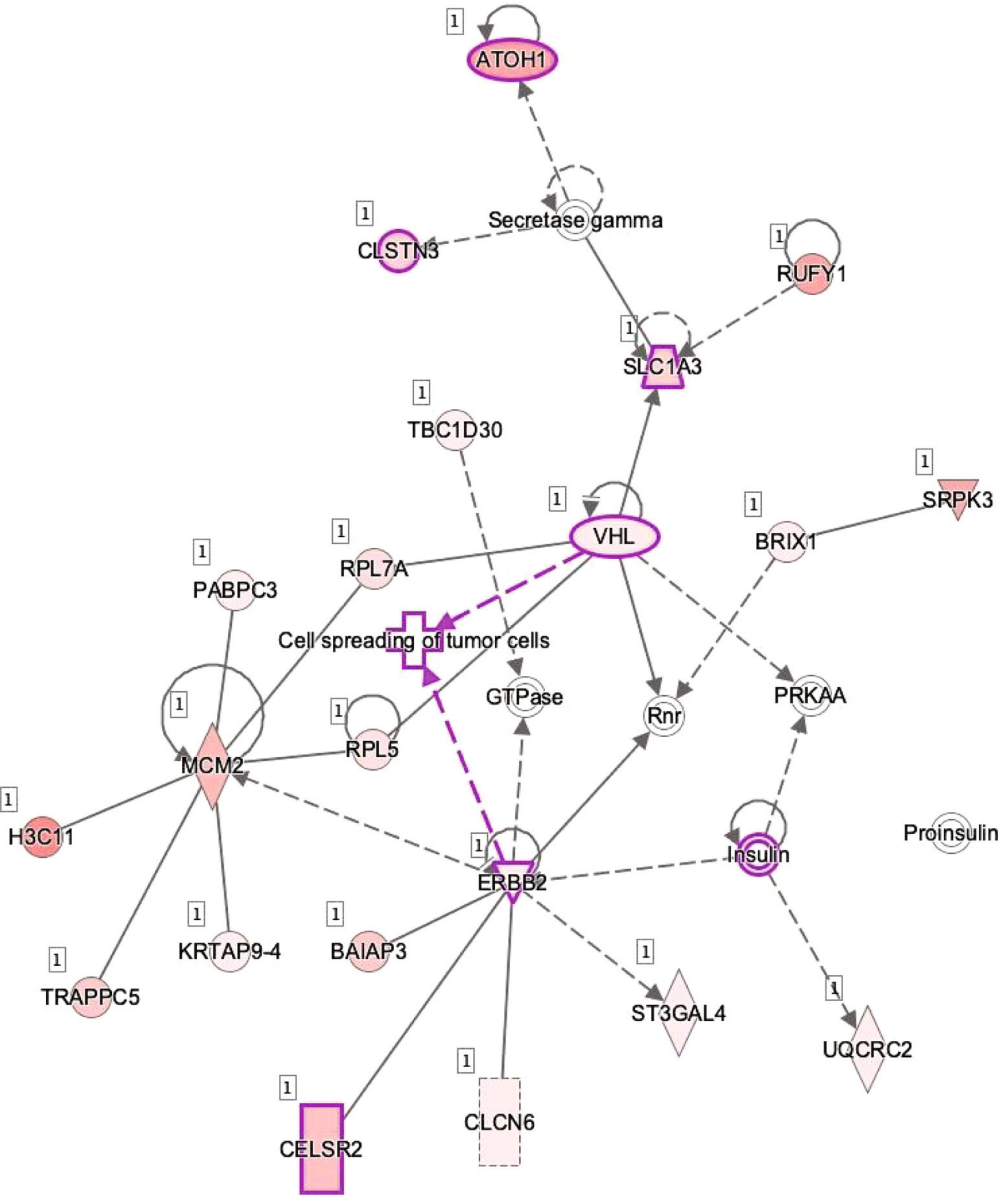
VHL gene, cell cycle, and cell morphology. This figure represents a novel regulatory network identified by IPA involving the VHL gene. Insulin as predicted to be upregulated in our dataset may be involved in the regulation of spreading of tumor cells.

Furthermore, pathways are enriched in genes for molecular and cellular function, including cellular development, movement, growth, and proliferation. Novel regulatory networks involving the VHL gene, cell cycle, and cell morphology were identified (Figure 10). Metabolic reprogramming, in this case by elevated glucose, could precipitate epigenetic modifications towards cancer [22].

**Figure 11:**
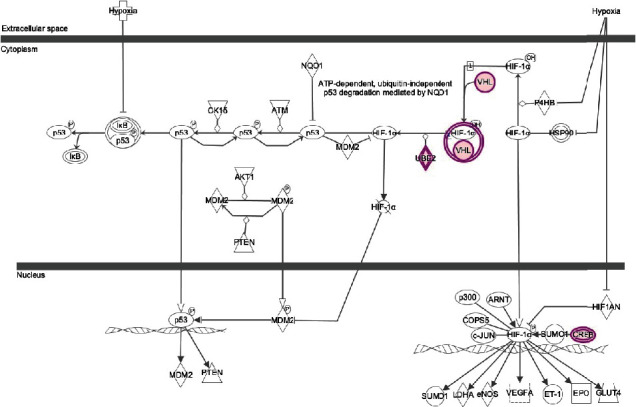
Hypoxia signaling in the cardiovascular system. This figure represents the hypoxia signaling in the cardiovascular system pathway as the main signaling pathway downregulated with a significant *p* value (*p* value = 2.13*E* − 02) caused by glucose treatment.

Therefore, a wide variety of networks and pathways were affected by an increased glucose concentration. One of the top diseases and disorders found by IPA is cancer (*p* value range from 1.35*E* − 02 to 1.95*E* − 25) with 339 molecules affected. Moreover, hypoxia signaling in the cardiovascular system pathway, including VHL, is involved (Figure 11).

**Figure 12:**
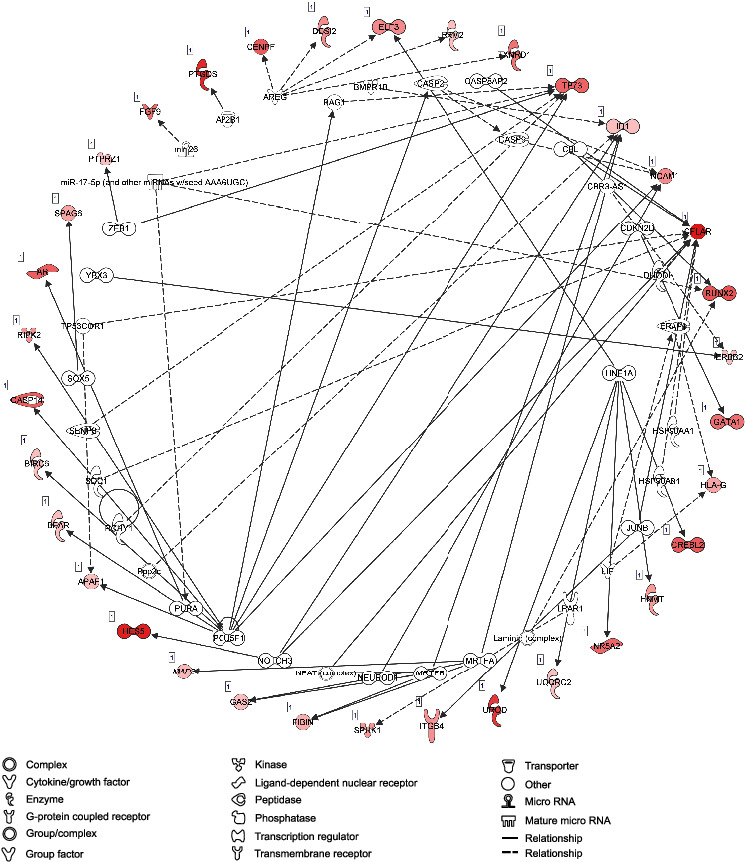
MRTFA and AREG with others identified as upstream regulators. This figure shows the many upstream regulators with predicted significant differential regulation resulting from high glucose treatment based on the hypermethylated or hypomethylated states of their downstream targets, identified by IPA upstream regulator analysis. For example, MRTFA is myocardia-related transcription factor with *p* value = 2.07*E* − 03 and AREG is an autocrine growth factor and a mitogen for astrocytes, Schwann cells, and fibroblasts with *p* value = 3.24*E* − 03. Other significant upstream regulators with predicted differential regulation include NOTCH3 with *p* value = 3.77*E* − 03, POU5F1 with *p* value = 4.06*E* − 03, and HNFIA with *p* value = 4.93*E* − 03. As described in our previous paper [16], a dashed line means indirect interaction, a continuous line means direct interaction, a line with an arrow means “acts on”, and a line with a bar at the end means “inhibits”. The relationships between molecules represented in the figure are based on effects reported in the literature, and the color-coding in the legend is used for correlation of known relationships with observed gene expression effects resulting from glucose treatment.

**Figure 13:**
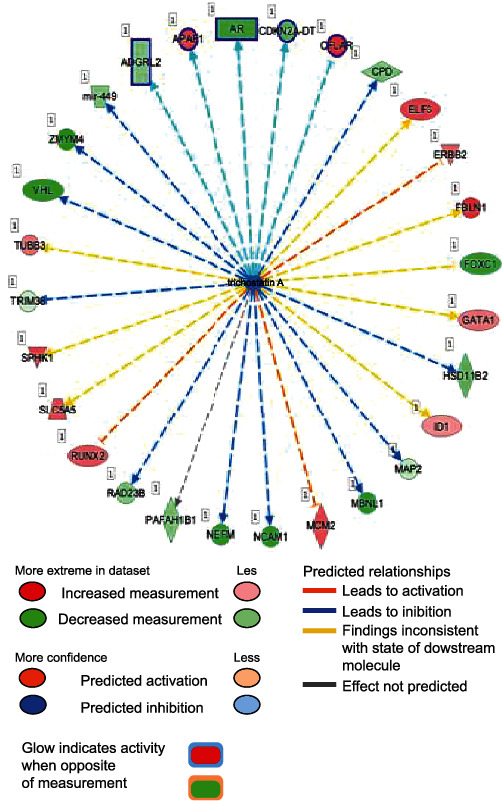
Trichostatin A. This molecule has a role in cell expression in apoptosis, acetylation, growth, transcription, activation, proliferation, binding, differentiation, and cell death. Trichostain A is at the center, and the dotted lines with arrows indicate downstream target genes that are upregulated (red) or downregulated (green) by differential methylation as indicated in our dataset. The meaning of line and arrow style is the same as for Figure 10.

**Figure 14:**
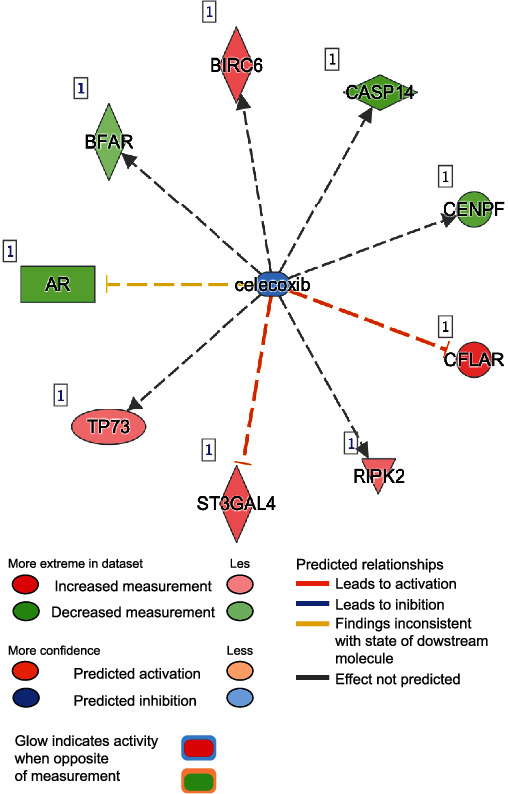
Celecoxib. This molecule has a role in cell expression in apoptosis, proliferation, growth, phosphorylation, cell death, survival, G1 phase, and activation. Celecoxib is at the center, and the dotted lines with arrows indicate downstream target genes that are upregulated (red) or downregulated (green) due to differential methylation as indicated in our dataset. The meaning of line and arrow style is the same as for Figure 10.

We next determined the main upstream regulators, including molecules, predicted for differential regulation based on the hypermethylated or hypomethylated state of their downstream targets (Figure 12) and found that high glucose concentration surprisingly correlated with the molecules trichostatin A (*p* value = 1.65*E* − 04) (Figure 13) and celecoxib (*p* value = 2.06*E* − 04) (Figure 14). This may be because trichostatin A affects the secretion pathway of beta cells and celecoxib may affect Cox-2 expression in DM.

Other significant upstream regulators with predicted differential regulation include PF-04928473 (*p* value = 2.11*E* − 04), doxorubicin (*p* value = 2.37*E* − 04), camptothecin (*p* value = 2.95*E* − 04), PC-SPES (*p* value = 6.16*E* − 04), MRTFA (*p* value = 2.07*E* − 03), AREG (*p* value = 3.24*E* − 03), NOTCH3 (*p* value = 3.77*E* − 03), POU5F1 (*p* value = 4.06*E* − 03), and HNFIA (*p* value = 4.93*E* − 03).

Out of the upstream regulators, we found five epigenetic enzymes with significant differential methylation, including DHODH, HSP90AA1, RCHY1, SDC1, and HSP90AB1 (Supplement 4, https://docs.google.com/spreadsheets/d/1AL4RfE4s6pXee_l3VtntmJOuxiPoF3N_/edit#gid=1482670607).

### 3.2. Diseases and Bio Functions

High glucose concentration impacted various cellular pathways including organismal injury and abnormalities. We identified 177 genes that were hypermethylated. Likewise, the major genes involved in these pathways are ANXA1, RPL5, HAL-G, MAP3K1, and NEDD4L. On the other hand, 379 genes were hypomethylated; for instance, SLC5A5, ID1, and SPHK1. These genes along with others were altered in (1) cellular development, growth, proliferation, and cancer; (2) gastrointestinal disease, inflammatory disease, and response; (3) cell death, survival, embryonic development, organismal injury, and abnormalities; (4) cell-to-cell signaling and interaction, hematological system development and function, and immune cell trafficking; and (5) cell cycle and ophthalmic disease (Supplement 5, https://docs.google.com/spreadsheets/d/1dx1aDfZTWEsROtmGMUjnILmYDda5qmSX/edit#gid=1594460684).

**Figure 15:**
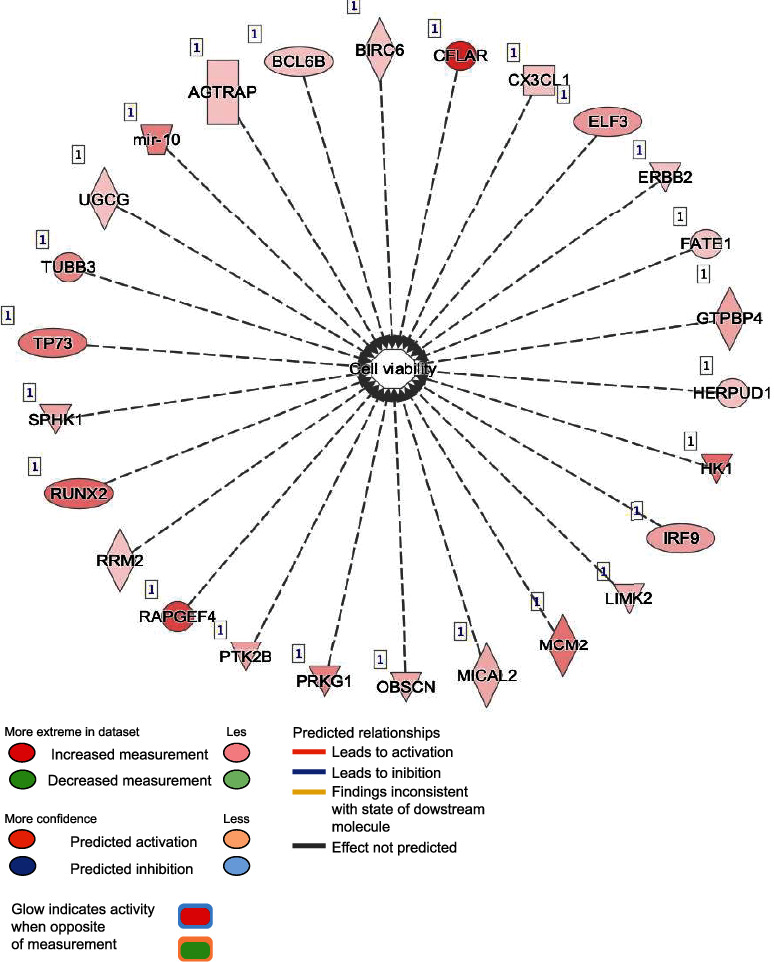
Cell viability. This figure represents many genes which may contribute to cell death and viability. Fifteen genes in the network were associated with cell death and cell survival with three of them being CFLAR, CX3CL1, and ERBB2 which were associated with survival of microglia network.

In the cell death and survival network, 55 genes were linked with necrosis, apoptosis, cell survival, and viability (Figure 15).

CFLAR, ERBB2, ID1, ITGB4, OBSCN, and TP73 play a role in the cell death of breast cell lines, while APAF1, DES12, SPHK1, and miR-10 are involved in apoptosis of lung cell lines. Also, RAPGEF4 and UGCG contribute to cell death of ovarian cell lines.

These results were interesting because of the known effects of high glucose on tissue and organ development. Also, the data points to potentially new targets that can be further explored in the future.

The main physiological systems affected by high glucose included tissue development, with 5 genes involved in megakaryocytopoiesis (*p* value = 3.32*E* − 02), 5 genes related to tubulation of vascular endothelial cells (*p* value = 2.18*E* − 02), 4 genes related to formation of muscle (*p* value = 3.53*E* − 02), and 3 genes related to cardiocentesis (*p* value = 9.89*E* − 03). Additionally, cardiovascular development and function was affected, with 9 genes involved in migration of vascular endothelial cells (*p* value = 4.69*E* − 03) and 4 genes related to permeability of endothelial cells (*p* value = 1.07*E* − 02).

## 4. Discussion

We have previously outlined how epigenetics addresses the relationship between genes, environment, and disease development [5]. Epigenetic state is affected by many factors such as age, lifestyle, family history, and disease status [2]. Here, we aimed to confirm the hypothesis that epigenetic changes in pancreatic beta cells may contribute to the etiology of DM via epigenetic modifications [5]. The results show that significant epigenetic changes are induced in pancreatic beta cells by high glucose, resulting in hundreds of genes being hypomethylated or hypermethylated. Higher glucose levels can cause genome-wide DNA methylation alterations in multiple genes that are predicted to impact signaling pathways and/or physiological systems, some of which we describe as follows.

### 4.1. Tissue and Organ Development

In terms of the top physiological systems affected according to IPA, 47 functions related to the reproductive system and function. Androgen deficiency predisposes to metabolic syndrome and T2D in men, while conversely in women, androgen excess is predisposing [23]. Androgens regulate many aspects of cellular metabolism, protein folding, and secretion pathways. The androgen receptor (AR) is downregulated by high glucose levels. Methylation of the AR gene could lead to many diseases, e.g., cancer and developmental disorders [24]. Previous studies have confirmed androgen regulation of many genes. Many of the genes regulate signal transduction and cell cycle and play an essential role in cellular protein trafficking.

Moreover, most of them are involved in the regulation of transcription and energy metabolism. For instance, androgens have a role in promoting survival and growth of prostatic epithelial cells. It has also been found by a previous microarray study that androgens regulate genes related to seminal fluid production [25]. There is a bidirectional interaction between DM and androgen depletion in men; a man diagnosed with prostate cancer might develop T2D and deficiency in testosterone hormone production. Conversely, testosterone deficiency impacts the development of obesity, insulin resistance, hypogonadism, and metabolic syndrome [23]. While in women with polycystic ovary syndrome, increased testosterone levels subsequently result in impaired glucose tolerance, beta cell dysfunction, and oxidative stress [26]. These studies and ours suggest that androgen hormone alterations in T2D might be epigenetic at source and at least partly responsible for reproductive system dysfunction.

### 4.2. Signaling Pathways: Molecular and Metabolic Interference

Primary pathways such as sulfide oxidation IV signaling, mitochondrial L-carnitine shuttle signaling, neuronal nitric oxide synthase (nNOS) signaling in skeletal muscle cells, and hypoxia signaling in the cardiovascular system were downregulated. The sulfide oxidation pathway regulates angiogenesis, cardioprotection, cell proliferation, neural development, apoptosis, and prevention of oxidative stress [27]. Also, it participates in the relaxation of blood vessels [28]. Moreover, it plays a role in inflammatory modulation and the production of reactive oxygen species. On the other hand, accumulation of H_2_S in the nervous system causes an increase in serotonin and a decrease in norepinephrine, glutamate, GABA, and aspartate [28]. nNOS signaling in skeletal muscle cells, nNOS phosphorylation, insulin stimulation, nitric oxide production, and GLUT4 translocation have all been reduced by inhibition of either nNOS or Akt2 [29].

Moreover, with a complete knockout of nNOS, in muscle strips from mice, insulin could not stimulate glucose uptake. The other signaling pathway of nNOS, when AMP-activated protein kinase (AMPK) is inhibited, prevented the activation of hydrogen peroxide (H_2_O_2_) and phosphorylation of nNOS, which leads to reducing NO production and significant attenuation of GLUT4 translocation [28]. Subsequently, nNOS is a common mediator of glucose uptake in both pathways, so the activation of both insulin and H_2_O_2_ signaling pathways will converge on nNOS [29].

Mitochondrial function is needed to produce ATP, and mitochondria are critical regulators of glucose to stimulate insulin secretion. During the past two decades, it has been found that mitochondrial dysfunction impacts DM, especially T2D, and involves many mechanisms, such as obesity, insulin resistance, pancreatic *β*-cell dysfunction, and vascular complications [30]. Another interesting finding in the data is the involvement of pathways for hypoxia signaling in the cardiovascular system. It will be important to integrate the role of hypoxia-inducible factors in DM with epigenetics [31].

### 4.3. Apoptosis and Death Receptors

Many studies suggest that endothelial damage and dysfunction is one of the early steps in the development of vascular complications. There is a close correlation between hyperglycemia and abnormalities in endothelial function and morphology [32]. Furthermore, high glucose levels increase DNA damage, affect the cell cycle, delay endothelial cell replication, and cause cell death in cultured human endothelial cells. Also, cell death of retinal microvascular cells leads to diabetic retinopathy. Apoptosis in mammals may be initiated by two different pathways that ultimately converge into a common pathway, resulting in effector enzyme caspase activation [32]. We identified four genes from the upstream regulators belonging to the caspase family of proteins: CASP2, CASP9, and CASP14, which are peptidases, and CASP8AP2, which is a transcription regulator associated with cell death survival, all differentially methylated by high glucose. Recent studies have shown that high glucose causes decreased mitochondrial membrane potential and the release of cytochrome c in human umbilical vein endothelial cells. Also in high glucose, gene expression levels of the death receptors TNF-R1 and Fas were increased [32]. Activation of caspases ultimately leads to cell disintegration [33].

### 4.4. Oxidative Stress

Oxidative stress affects cellular metabolism. Endoplasmic reticulum (ER) stress occurs when proteins are misfolded during biosynthesis. As a result of the unfolded protein response in beta cells, insulin transcription and translation are reduced, and inflammation and apoptosis initiated, while protein misfolding causes the production of reactive oxygen species (ROS) [34, 35]. Thus, beta cell dysfunction involving oxidative and ER stress, impaired secretory function, beta cell apoptosis, and islet inflammation might progressively cause T2D. Accumulation of *β*-amyloid, which is one feature of human diabetic islets, has been connected to oxidative stress and apoptosis without ER stress [34, 35]. We found one gene, SUOX, with significant methylation (*p* value = 2.04*E* − 03) that has a role in oxidoreductase activity, protein binding, and sulfite oxidase activity. Oxidative stress is considered a crucial feature of beta cell dysfunction in T2D and of beta cell depletion in type 1 diabetes. Also, oxidative stress has many harmful effects on beta cell function, including suppression of insulin transcription. However, some ROS production is needed for signaling within beta cells and normal beta cell function [34, 35].

### 4.5. Study Limitations

Finally, there are some limitations with this study. Firstly, only beta cells were used in the experiment, but *in vivo*, pancreatic islets contain other cells -alpha and others- which could affect the cells' behavior *in vivo*. Secondly, we have not yet looked at reversibility of the epigenetic changes, and it is possible that some of these changes are reversible if the glucose level is normalized. This will be the focus of future experiments. Lastly, *in vivo* glucose levels fluctuate, but in our study, they were constant and unchanging. We will try to replicate more “lifelike” glucose fluctuations in future experiments.

## 5. Conclusions

In this study, we wanted to explore the potential of high glucose levels to cause epigenetic changes in human beta cells in an initial trial experiment. We used human genome-wide promoter methylation analysis to identify alterations caused by a high glucose concentration. MeDIP-Chip microarray analysis revealed that many genes were hypermethylated or hypomethylated. We have highlighted canonical pathways and mechanistic networks that are related to DM. The networks that were found as a result of this analysis may provide insight into genes that can be further studied. They may provide valuable knowledge pointing to epigenetic changes in pancreatic beta cells in the etiology of DM. We view this paper as the beginning of a more extensive investigation into the epigenetic mechanisms and potential etiology of DM.

A future investigation will also include metformin. Metformin might change the epigenetic networks at least partially back to normal and influence and stimulate beta cells to increase insulin hormone secretion. It will be important to explore this hypothesis further in future studies: to better understand the epigenetic changes in pancreatic beta cells in DM and also understand their reversibility.

## Data Availability

Supplementary files are available on Google Drive at: https://drive.google.com/drive/u/0/folders/1J80zzwrZb6S7CgHVvzkIXR-Av6Ij-1nf.

## Supplementary Materials

Supplementary Materials Supplement 1a: data was processed following the protocol of our previous paper [16]. Table with a list of significantly differentially methylated peaks (*p* < 0.01) that lie within 2000 base pairs of the transcription start site of a gene, generated using the initial raw data. All coordinates were transformed from hg18 to hg38, and the genes were reannotated. Peaks were remapped to the latest genome (hg38) and refiltered according to distance from the nearest gene TSS. The “Direction” column indicates which way the differential methylation goes (hypermethylated in treated or hypomethylated). The “Peak differential methylation” column (col M) shows the average difference in methylation level between treated and untreated (positive values are higher methylation in treated compared to untreated; negatives are lower). There are more peaks hypomethylated in treated than hypermethylated (285 hypomethylated peaks vs. 193 hypermethylated peaks). Each peak lies within 2000 base pairs of one or more genes. The list of genes is in column C and the distance of their TSS from the middle of the peak is in column L. Positive numbers indicate that the peak middle is upstream of the TSS, and negative numbers indicate that the peak middle is downstream. Supplement 1b: means for all of the significant peaks. Supplement 2: data was processed following the protocol of our previous paper [16]. Table with list of significantly differentially methylated peaks (*p* < 0.01) that lie within 2000 base pairs of the transcription start site of a gene, used as raw data for IPA. Hypomethylated gene promoters with significantly differentially methylated peaks were assigned positive signs, and hypermethylated gene promoters were assigned negative signs, to correlate with upregulation or downregulation of gene expression, respectively. Supplement 3: data was processed following the protocol of our previous paper [16]. Table listing all genes linked to mechanistic networks and canonical pathways. Information regarding gene/molecule and their respective ENTREZ Gene IDs for human, mouse, and rat are specified. Supplement 4: data was processed following the protocol of our previous paper [16]. Table listing significant upstream regulators with predicted differential regulation. From these upstream regulator's gene, five epigenetic enzymes were found with significant differential methylation. Supplement 5: data was processed following the protocol of our previous paper [16]. An all-inclusive list of organismal injury and abnormalities-related genes based on significant genes from our dataset as identified by IPA analysis of high glucose treated human cells. Each function with *p* value and activation *z* score, genes/molecule names, and total number of genes/molecules with predicted differential regulation from high glucose treatment are listed.
