# Supplementary material for: Epigenetic Changes Induced by High Glucose in Human Pancreatic Beta Cells
Source: J Diabetes Res. 2023 Feb 13;2023:9947294. doi: 10.1155/2023/9947294 (PMC9940985; doi:10.1155/2023/9947294)
Supplement: Supplementary Materials — Supplement 1a: data was processed following the protocol of our previous paper [16]. Table with a list of significantly differentially methylated peaks (p < 0.01) that lie within 2000 base pairs of the transcription start site of a gene, generated using the initial raw data. All coordinates were transformed from hg18 to hg38, and the genes were reannotated. Peaks were remapped to the latest genome (hg38) and refiltered according to distance from the nearest gene TSS. The “Direction” column indicates which way the differential methylation goes (hypermethylated in treated or hypomethylated). The “Peak differential methylation” column (col M) shows the average difference in methylation level between treated and untreated (positive values are higher methylation in treated compared to untreated; negatives are lower). There are more peaks hypomethylated in treated than hypermethylated (285 hypomethylated peaks vs. 193 hypermethylated peaks). Each peak lies within 2000 base pairs of one or more genes. The list of genes is in column C and the distance of their TSS from the middle of the peak is in column L. Positive numbers indicate that the peak middle is upstream of the TSS, and negative numbers indicate that the peak middle is downstream. Supplement 1b: means for all of the significant peaks. Supplement 2: data was processed following the protocol of our previous paper [16]. Table with list of significantly differentially methylated peaks (p < 0.01) that lie within 2000 base pairs of the transcription start site of a gene, used as raw data for IPA. Hypomethylated gene promoters with significantly differentially methylated peaks were assigned positive signs, and hypermethylated gene promoters were assigned negative signs, to correlate with upregulation or downregulation of gene expression, respectively. Supplement 3: data was processed following the protocol of our previous paper [16]. Table listing all genes linked to mechanistic networks and canonical pathways. Informat [file 9947294.f1.pdf]

Supplementary data can be found at the following links:

Main Supplementary Data Folder

https://drive.google.com/drive/u/0/folders/1J80zzwrZb6S7CgHVvzkIXR-Av6Ij-1nf

Supplement 1a

https://docs.google.com/spreadsheets/d/10npdt2_UEH9T3gX3d3qZIkm07mf5mtJf/edit#gid=190629373
2

Supplement 1b

https://docs.google.com/spreadsheets/d/1_SKHu_rP3wDacDzDqUUS_zN88XaEVhUx/edit#gid=1273186
519

Supplement 2

https://docs.google.com/spreadsheets/d/17tlE8xz3xbaNPrI9Iz18YZXOmjF4qlpt/edit#gid=407977637

Supplement 3

https://docs.google.com/spreadsheets/d/1vV9kzpqkS4Zoc_4Hmi3lMrXrvZhpw19v/edit#gid=386233314

Supplement 4

https://docs.google.com/spreadsheets/d/1AL4RfE4s6pXee_l3VtntmJOuxiPoF3N_/edit#gid=1482670607

Supplement 5

https://docs.google.com/spreadsheets/d/1dx1aDfZTWEsROtmGMUjnILmYDda5qmSX/edit#gid=1594460
684
